# Comprehensive in silico allergenicity assessment of novel protein engineered chimeric Cry proteins for safe deployment in crops

**DOI:** 10.1186/s12896-017-0384-z

**Published:** 2017-08-02

**Authors:** Maniraj Rathinam, Shweta Singh, Debasis Pattanayak, Rohini Sreevathsa

**Affiliations:** ICAR-National Research Centre on Plant Biotechnology, LBS Centre, Pusa Campus, New Delhi, 110012 India

**Keywords:** Allergenicity, *Bacillus thuringiensis*, Cry proteins, Food crops, Transgenics, Insect resistance

## Abstract

**Background:**

Development of chimeric Cry toxins by protein engineering of known and validated proteins is imperative for enhancing the efficacy and broadening the insecticidal spectrum of these genes. Expression of novel Cry proteins in food crops has however created apprehensions with respect to the safety aspects. To clarify this, premarket evaluation consisting of an array of analyses to evaluate the unintended effects is a prerequisite to provide safety assurance to the consumers. Additionally, series of bioinformatic tools as in silico aids are being used to evaluate the likely allergenic reaction of the proteins based on sequence and epitope similarity with known allergens.

**Results:**

In the present study, chimeric Cry toxins developed through protein engineering were evaluated for allergenic potential using various in silico algorithms. Major emphasis was on the validation of allergenic potential on three aspects of paramount significance viz., sequence-based homology between allergenic proteins, validation of conformational epitopes towards identification of food allergens and physico-chemical properties of amino acids. Additionally, in vitro analysis pertaining to heat stability of two of the eight chimeric proteins and pepsin digestibility further demonstrated the non-allergenic potential of these chimeric toxins.

**Conclusions:**

The study revealed for the first time an all-encompassing evaluation that the recombinant Cry proteins did not show any potential similarity with any known allergens with respect to the parameters generally considered for a protein to be designated as an allergen. These novel chimeric proteins hence can be considered safe to be introgressed into plants.

**Electronic supplementary material:**

The online version of this article (doi:10.1186/s12896-017-0384-z) contains supplementary material, which is available to authorized users.

## Background

Agricultural biotechnology has gained enormous thrust since the latter half of twentieth century. One of the major revolutions has been the successful adoption of transgenic technology both in lab as well as in land. Development of insect resistant plants was given primary impetus as one of the primary utilities of the technology. There has been a continued quest for search of novel genes and technologies to confer insect resistance to crop plants. *Bacillus thuringiensis-*mediated insect management has been in practice since 1938 [1]; alongside its usefulness as a biopesticide, the crystalline protein genes (*cry*) of the bacterium have been used as insecticidal proteins [2]. Despite the identification of a large number of *cry* genes globally, the search for novel genes is still on [3]. However, the success of *cry* gene–based transgenic insect resistant crops has demonstrated the potential of these proteins and the adoption of the technology worldwide [4].

Among the various classes of *cry* genes, the *cry1* series of genes are generally effective against Lepidoptera and are species specific [1, 5]. In spite of the superiority in the efficacy of the genes identified thus far, the need of the hour is towards development of novel toxins with broad spectrum insecticidal activity due to the growing concern about resistance development in the insects to these toxins. This has resulted in increased focus on the identification and development of novel chimeric Cry proteins using technologies like protein engineering through domain swapping. Exploitation of these hybrid or chimeric *Bt* genes is proposed to be advantageous in providing good and long term protection against a range of pests [5–9]. The concept of chimeric proteins was developed based on the hypothesis that combining domains of validated and effective Cry proteins would not only result in improved efficacy of the resultant hybrid but would also delay the onset of resistance towards these proteins in the target insects. Several studies in our laboratory have demonstrated the efficacy of these chimeric genes that were synthesised by domain swapping of proven effective genes [8, 10, 11]. Nonetheless, it is important to analyse concomitantly whether such chimeric genes are safe and non-allergenic for consumption prior to commercialization.

Allergenicity through food and feed is one of the primary concerns to mankind as food allergy due to various substances is increasing in both adults and children [12]. With respect to transgenic food crops, it is essential to have clarity that the food crops are being engineered with proteins that do not cause any allergic symptoms in the consumers. Hence, demonstration that *cry* genes are non-allergenic is one of the ways to improve acceptance of transgenic food crops for insect resistance. Bioinformatics has come in as one of the quickest means to demonstrate whether a protein is allergenic or not based on sequence and epitope similarities. There are many reliable softwares and tools that unambiguously predict whether or not a given protein is allergenic [13, 14] based on sequence similarity, presence of allergenicity related linear motifs like IgE epitopes and physico-chemical properties of amino acids. Nevertheless, it is essential to use more than one software for explicit proof due to the increased probability of occurrence of false positives if the search is narrowed [15]. There exists a plethora of information about in silico analysis of various Cry proteins being used in the development of transgenic crops [16, 17]. However, there is no information available about the allergenicity of novel chimeric Cry proteins developed through protein engineering and domain swapping. The present study is the first of its kind to demonstrate non-allergenic potential of selected chimeric Cry proteins using a comprehensive in silico and supportive in vitro analyses.

## Methods

### Source of genes for in silico analysis

*Cry* genes with proven efficacy against lepidopteran pests were selected for protein engineering through domain swapping. Sequences of thus developed novel chimeric *cry* genes (Table 1) were converted to protein sequences in FASTA format and used for bioinformatics analysis.

**Table 1:** Novel protein engineered chimeric *cry* genes used in the study

Sl. No	Chimeric genes	Parental genes/Domains	Target insects
1	*Cry1Aabc*	*Cry1Aa*(D-I) *Cry1Ab*(D-II) Cry1Ac(D-III)	Lepidoptera
2	*Cry1AcF*	*Cry1Ac*(D-I-II) *Cry1F*(D-III)	Lepidoptera
3	*Cry1AbBaBa*	*Cry1Ab*(D-I) *Cry1Ba*(D-II-III)	Lepidoptera/Diptera
4	*Cry1BaBaAb*	*Cry1Ba*(D-I-II) *Cry1Ab*(D-III)	Lepidoptera/Diptera
5	*Cry1AbBaAb*	*Cry1Ab*(D-I) *Cry1Ba*(D-II) *Cry1Ab*(D-III)	Lepidoptera/Diptera
6	*Cry1AcJAc*	*Cry 1Ac*(D-I) *Cry1J*(D-II) *Cry1Ac*(III)	Lepidoptera
7	*Cry1AaIa5Ia5*	*Cry1Aa* (D-I) *Cry1Ia5*(D-II-III)	Lepidoptera
8	*Cry1AaB*	*Cry1Aa*(D-1) *Cry1B*(D-II-III)	Lepidoptera

### Assessment of homology between query and database protein using full length FASTA search

#### Allergen online database 17.0

Allergen online Database (AOL) is a peer reviewed open access database maintained by Food Allergy Research and Resource Program (FARPP) and was introduced by Department of Food Science and Technology at University of Nebraska, Lincoln. The database is functional from 2007 with constant updating and has a list of 2035 allergenic and putative allergenic protein sequences (http://www.allergenonline.org/AllergenOnlineV17.pdf) and 808 taxonomical protein groups. BLOSUM 50 score matrix is used in the database to predict homology between query and database proteins. All the database entries in AOL are linked to the sequences in National Centre for Biotechnology Information (NCBI) of National Institute of Health (NIH). In the present study, full length FASTA3 search was carried out for the respective chimeric proteins using the updated database (Version#17; January 2017) with default settings; percentage similarity more than 70% was considered as putative allergen and below 50% not likely to be an allergen [18]. E-score was generated based on the similarity in protein sequence or functional analogy in amino acids. Low degree of similarity between allergenic sequences present in the database reflected high E value.

### Structural database of allergenic proteins (SDAP)

SDAP is an online free web server store that stocks structural and sequence information of known allergens from international union of immunological societies (IUIS) and is linked to various protein servers – Protein Data Base (PDB), SWISS-PROT, Protein Information Resource (PIR), GenBank. This database has information on 1526 allergens and isoallergens. Full length FASTA alignment was performed in our study for all the Cry proteins against allergenic proteins in SDAP and aligned by FASTA 3.45. Query with E-value below 0.1 was considered as a putative allergen.

### Assessment of homology between query and database proteins using 80mer sliding window search

The sequences of the selected chimeric Cry proteins were evaluated by FASTA search for the alignment with the listed sequences in the database. For this, each of the protein sequences was aligned in an online 80mer window in 1–80 and 2–81 sliding fashion. Any protein with >35% sequence identity as a default threshold value with known allergens was presumed to produce cross reactivity with IgEs.

### Assessment of homology between query and database proteins using DELTA-BLAST

Domain Enhanced look-up Time Accelerated (DELTA)-BLAST [19] predicts remote homology through blast search, providing comparatively more homologous alignment. It performs long presumed homologous alignment with conserved domains to construct its Position Specific Score Matrix (PSSMS) and facilitates efficient search compared to other blast search engines [20]. As entrez query limits the search based on the keyword entered, the selected *cry* gene sequences were run on DELTA-BLAST with Entrez Query using keyword “Allergen”. Best alignment was selected based on the least E-value (E-value <0.01).

### Assessment of allergenic properties of *cry* genes through machine learning language

#### Algpred 1.0

Algpred (www.imtech.res.in/ragava/algpred/) [21] is a web server that predicts the allergenic protein and maps IgE epitopes on the protein. Four different tools are used to predict allergenic proteins – Firstly, it predicts the allergen by using Support Vector Machine (SVM) by taking input as amino acid and dipeptide composition of proteins. Secondly, it adopts MEME/MAST tool for motif-based allergenic protein prediction. Thirdly, it aligns against 2890 Allergen Representative Peptides (ARPs) and fourthly, it predicts the known IgE epitopes on the query protein sequence. This forms a hybrid approach of high accuracy for the prediction of allergenic potential of proteins. This hybrid approach to identify the allergenic potential was performed for all the selected chimeric Cry proteins to analyse for the presence of IgE epitopes.

#### AllerTop 1.0

AllerTop (www.pharmfac.net/allertop) [22] is another web based server that was used in the study for validation of the chimeric proteins. It is an alignment-free server that predicts allergenicity of proteins based on the physicochemical properties of amino acids present in the protein sequence. Firstly, it uses Z-descriptors (Z_1_- hydrophobicity, Z_2_ - molecular size and Z_3_-polarity) to represent amino acids in peptide sequence and later uses Auto and Cross-Covariance (ACC) transformation for conversion of peptide sequences to uniform vectors. It eventually uses “*k* Nearest Neighbors (*k*NN)” method that predicts the route of exposure based on three nearest neighbors of known allergens to distinguish between allergenic and non allergenic proteins.

#### In silico assessment of pepsin digestion sites in the chimeric proteins

In silico protein digestion test was performed by ExPASy-peptide cutter server (http://web.expasy.org/peptide_cutter/) [23]. Protein sequences of two of the chimeric proteins, Cry1AcF and Cry1Aabc were subjected to in silico pepsin digestion with recommended pH conditions (pH 1.5 and 2.0). The assay pH was described by FAO/WHO [24].

### In vitro assessment of the chimeric proteins for allergenic potential

#### Expression and purification of Cry1Aabc and Cry1AcF proteins from *E. coli*

The genes *cry1AcF* and *cry1Aabc* were cloned separately in the expression vector pET-29a and mobilized into *E. coli* strain BL-21. For protein expression, 6 ml of an overnight grown culture was added to a 200 ml LB medium and incubated with vigorous shaking at 37 °C. At an OD_600_ of 0.6–1.0 of the culture, protein expression was induced with 1 mM IPTG at 37 °C for 4 h. Cells were later collected by centrifugation and resuspended in 20 ml buffer A (50 mM carbonate buffer, 150 mM NaCl, 2 mM PMSF and 10 mM βME) followed by sonication at 4 °C for 30 cycles with each cycle consisting of 10 s of sonication and 20 s of resting time. The pellets were later resuspended in buffer B (50 mM carbonate buffer, 300 mM NaCl, 2 mM PMSF, pH 9.0). A ‘Protein A gravity flow column’ (Biorad) was washed with 20% ethanol and was loaded onto 3 ml Ni-NTA beads (Probond resin, Novex by Life technologies) and the solid matrix (50% slurry in 20% ethanol) was equilibrated upto 2 ml mark. The column was further equilibrated with buffer B and 10 mM imidazole was added prior to protein loading. After the flow through was eluted, the slurry was washed twice with 25 ml buffer B (wash buffer I) and twice with wash buffer II (buffer B + 20 mM imidazole). Later, the protein was eluted in 1.5 ml collection tubes following the addition of 4 ml elution buffer (buffer B + 300 mM imidazole). Concentration of the purified proteins was calculated and used for further experiments.

### Thermal stability of the chimeric proteins

The purified Cry1Aabc and Cry1AcF proteins were dissolved in carbonate buffer (pH 9.5) at a concentration of 0.1 mg/ml in 1.5 ml microcentrifuge tubes and incubated at 100 °C for different time intervals i.e., 10, 30, and 60 min. The experiment was stopped by placing the tubes on ice after allotted time intervals and SDS sample buffer (50 mM Tris-HCl, 8% sucrose, 2% SDS, with 5% 2-mercaptoethanol, and 0.02% bromophenol blue) was added in each tube. Control sample was prepared with 0 min incubation of the proteins (kept at 4 °C) [25]. The proteins were further separated on SDS-PAGE (silver staining) and analysed by western blot.

### In vitro digestibility of the chimeric proteins with pepsin

The stability of the purified Cry1Aabc and Cry1AcF proteins was evaluated with purified porcine pepsin following the standardized procedure [26] with modifications. The purified proteins (0.1 mg/ml) were taken in simulated intestinal fluid (SIF) (Sigma, USA) with pH set at 1.2 and 2.0 [24]. Pepsin was added to the sample mixture at the concentration of 10 U/μg of test protein. The sample mixtures were incubated at 37 °C and taken out at the intervals of 0 s, 30 s, 60 s, 5 min, 10 min and 30 min. Control samples consisted of test protein in SIF buffer without pepsin with 5 min incubation, test protein in pepsin without SIF buffer with 5 min incubation and undigested intact proteins with incubation of 5 min at 37 °C. The digestibility was later evaluated by subjecting the assay mixtures to SDS-PAGE.

## Results and discussion

### Sequence homology-based allergenicity assessment of the chimeric proteins

Novel *cry* genes developed through protein engineering tools like domain swapping between different *cry* genes are being increasingly identified and synthesized for improved resistance management strategies. The present study, a first of its kind, demonstrated the non-allergenic potential of such protein engineered novel *cry* genes using an array of bioinformatic tools (diverse set of sequence and epitope-based algorithms) and in vitro analysis.

**Fig. 1:**
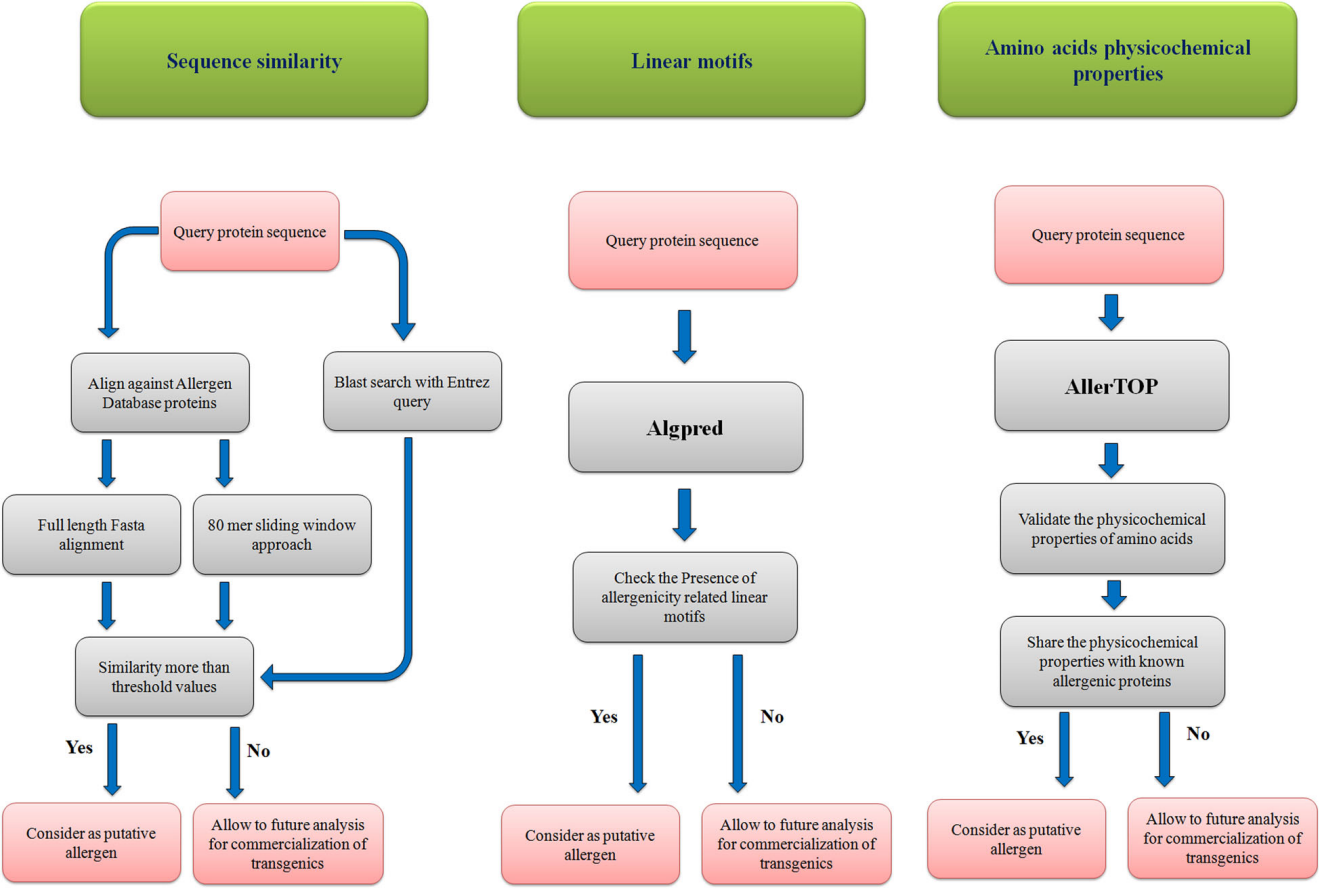
Protocol for sequence and epitope homology-based allergen prediction

As the prediction of allergenicity potential of any protein cannot be achieved following a single step analysis, a comprehensive strategy has been designed by FAO/WHO and Codex Alimentarius Commission (2003) (Fig. 1). In this investigation, full length FASTA search was performed on Allergen Online Database (AOL) with default settings and it was observed that the selected genes did not show any similarity between the proteins present in the database with expected E value <1.0; however, Cry1AcF and Cry1BaBaAb exhibited an E value below 1.0 (Table 2). It was observed that Cry1AcF showed 23.7% similarity against Gamma-Gliadin food allergen from bread wheat (*Triticum aestivum*) with 0.49 E-value whereas Cry1BaBaAb shared similarity with the food allergen gamma-gliadin precursor (26.8%) from bread wheat (*Triticum aestivum*) and Der f Mal f 6 allergen (24%) from *Dermatophagoides farinae* with E values 0.65 and 0.96 respectively. Results of full length FASTA search reflected that the recombinant proteins did not meet the suggested threshold value of 50% similarity [18] and served as an initial proof to demonstrate lack of homology with any of the known allergens.

**Table 2:** Summary of in silico analyses for assessment of allergenic potential of the chimeric cry proteins

Cry Proteins	Full length FASTA search				80mer sliding window search			NCBI DELTA-BLAST (% identity& E-value)		Algpred 1.0	AllerTop 1.0
	Allergen online 17.0 (% identity& E-value)		SDAP (% identity)		Allergen online 17.0 (% identity)	SDAP (% identity)					
Cry1Aabc	No matches with E score < 1.0		9.02%	Gly m Bd2 8 K, (BAB21619)	No matches with >35%	28.75%	Asp f 13 (P28296)	No matches with E score < 0.01		Non –Allergen	Probably Non - Allergen
			5.80%	Ana c 2 (BAA21849)		26.25%	Der f 1.0102 (2428875)				
			5.80%	Tri a gliadin (AAA34285)		27.50%	Alt a 2 (AAD00097)				
Cry1AcF	23.7%	Gamma-Gliadin (170738) E - 0.49	8.99%	Gly m Bd2 8 K (BAB21619)	No matches with >35%	27.50%	Hev b 1 (P15252)	17%	Pectate lyase/Amb allergen (EEU00825.1) E – 9e-07	Non –Allergen	Probably Non - Allergen
			5.78%	Tri a gliadin (AAA34285)		27.50%	Alt a 2 (AAD00097)	17%	Pectate lyase/Amb allergen (ACX76150.1) E – 2e-06		
			5.78%	Eur m 3 (O97370)		26.25%	Lig v 1 (O82015)	15%	Fibronectin type III domain protein (ACT03889.1) E – 6e-04		
Cry1AbBaBa	No matches with E score < 1.0		6.28%	Ani s11.0101(BAJ78220)	No matches with >35%	28.75%	Pis v 3.0101 (EF116865)	No matches with E score < 0.01		Non –Allergen	Probably Non - Allergen
			4.71%	Lig v 1(O82015)		26.25%	Pet c PR10 (CAA67246)				
						26.25%	Lig v 1 (O82015)				
Cry1BaBaAb	26.8%	Gamma-Gliadin precursor (1063270) E − 0.65	5.58%	Mala s 1 (Q01940)	No matches with >35%	32.50%	Mala s 1 (Q01940)	14%	Pectate lyase/Amb allergen (ABN54148.1) E – 8e-08	Non –Allergen	Probably Non - Allergen
	24.0%	Der f Mal f 6 allergen (37958141) E − 0.96	4.50%	Asp f 17 (CAA12162)		27.50%	Asp f 17 (CAA12162)	14%	Pectate lyase/Amb allergen (EEU00825.1) E – 7e-08		
			4.19%	Pis v 4.0101 (EF470980)		26.25%	Asp f 10 (CAA59419)				
Cry1AbBaAb	No matches with E score < 1.0		5.77%	Mala s 1 (Q01940)	No matches with >35%	32.50%	Mala s 1 (Q01940)	14%	Pectate lyase/Amb allergen (EEU00825.1) E – 9e-08	Non –Allergen	Probably Non - Allergen
			4.81%	Lig v 1 (O82015)		27.50%	Asp f 17 (CAA12162)	14%	Pectate lyase/Amb allergen (ABN54148.1) E – 1e-07		
			4.81%	Asp f 17 (CAA12162)		26.25%	Asp f 10 (CAA59419)				
Cry1AcJAc	No matches with E score < 1.0		7.62%	Gly m 6.0501 (Q7GC77)	No matches with >35%	32.50%	Api m 3.0101 (NP_00101337)	No matches with E score < 0.01		Non –Allergen	Probably Non - Allergen
			7.28%	Api m 3.0101 (NP_00101337)		30.0%	Dol m 1.0101 (Q06478)				
			5.79%	Eur m 3 (O97370)		28.75%	Gly m 1 (AAB09252)				
Cry1AaIa5Ia5	No matches with E score < 1.0		8.41%	Per a 3.0101 (Q25641)	No matches with >35%	26.5%	Lig v 1 (O82015)	No matches with E score < 0.01		Non –Allergen	Probably Non - Allergen
			6.23%	Sol i 1.0101 (AAT95008)		26.5%	Pol f 5 (P35780)				
			5.51%	Asp f 4 (O60024)		25%	Ana c 1 (AAK54835)				
Cry1AaB	No matches with E score < 1.0		6.21%	Ani s 11.0101 (BAJ78220)	No matches with >35%	27.50%	Alta2 (AAD00097)	No matches with E score < 0.01		Non –Allergen	Probably Non - Allergen
			5.	Car p papain (AAB02650)		26.50%	Ligv1 (O82015)				
			4.94%	Alt a 2 (AAD00097)		25%	Pen c 24 (AAR17475)				

SDAP revealed that all the proteins encoded by the chimeric *cry* genes showed minimal (not more than 10%) similarity with the allergenic proteins and were far below the set threshold level (Table 2). This indicated that the protein engineering had not introduced any unintended effects in the chimeric proteins and were thus safe to be integrated into food crops. Further supportive evidence was obtained when the sliding 80mer window search of the chimeric proteins did not show >35% similarity against any known allergen (Table 2) demonstrating that the search did not meet the criteria of codex for cross reactivity between the allergenic proteins and were thus safe and non-allergenic.

BLAST alignment was performed for the cry proteins with ENTREZ query limits “ALLERGEN”, so that the blast would align the query with known allergenic proteins. This blast is more sensitive for the protein–protein alignment and the frequency of occurrence of false positives is less compared to other blast search engines [19]. The search demonstrated that the chimeric proteins Cry1Aabc, Cry1AbBaBa, Cry1AcJAc, Cry1AaIa5Ia5 and Cry1AaB did not show any similarity with the known allergenic proteins with an E score of <0.01. Cry1AcF showed 17% similarity with a pectate lyase/Amb allergen from different sources like *Clostridium thermocellum* and *Fibrobacter succinogenes* with E-values of 9e-07 and 2e-09 respectively and 15% identity with Fibronectin type III domain protein from *Paneni bacillus sps* JDR-2 with E-value of 6e-04. Cry1BabaAb and Cry1AbBaAb showed 14% similarity with bacterial allergen - Pectate lyase/Amb allergen from *Ruminiclostridium thermocellum.* Further, the study demonstrated that all the query proteins did not shown more than 35% similarity with any of the allergenic proteins reiterating their safe nature as per the guidelines recommended by Codex Alimentarius Commission, 2003.

The sequence homology-based identification of similarity with allergenic proteins is the generally followed pipeline for assessment of cry proteins and this was unequivocally established with respect to the chimeric proteins of the present study. However, the major concern with genetic engineering of food crops is the possibility of it being resistant to gastric digestion, which is a key characteristic nature of food allergens. It is proposed that the food matrix thus could be protecting the allergens from digestion which will allow them to retain their native structure, making the validation of conformational epitopes very important. This aspect assumes significance and needs to be given a serious thought with respect to Genetically Modified (GM) foods. Therefore, assessment of allergenicity using in silico tools exploiting the conformational epitopes is very important.

### Allergenicity assessment of the chimeric proteins based on linear motifs and physicochemical properties of amino acids

In our study, analysis of the chimeric proteins based on the conformational epitopes was further authenticated by using two specific algorithms, Algpred and AllerTop for better clarity about the non-allergenic potential of the chimeric proteins. Algpred utilizes a hybrid approach and combines four steps of motif search involving SVM, MEME/MAST, IgE epitopes and Allergen-Representative peptides (ARPs) [27] as allergen-specific protein structures and motifs have been reported in a few families of proteins [28]. Clear evidence was obtained in the present study that the eight chimeric cry proteins did not share sequence similarity with IgE epitopes of known allergens (Table 2). In the same way, it was also demonstrated in the study that there was no sequence similarity of the chimeric protein genes with the collection of ARPs [27]. Identification of motifs occurring commonly in allergens but rarely in ordinary proteins is a very important aspect before designating a protein as a non-allergen. The absence of similarity with the ARPs, which are specific to allergens showed that the genes were safe for deployment in food crops.

Algpred and other local alignment tools recommended by codex commission predict the allergenic potential of any protein based on the sequence homology by considering linear epitopes with immunogenic properties. In contrast, it is an interesting fact that IgE binding B cell epitopes are not only linear but also conformational epitopes, and therefore share less homology with proteins [29]. It has been earlier reported that allergen specific patches in proteins are dominated by hydrophobic amino acids on the surface [30], leading to the inability of the alignment-based approaches to detect allergenicity in an unambiguous manner. This defines the need for prediction of allergenicity based on the physicochemical properties of amino acids like hydrophobicity, molecular size and polarity. Our study presents the utility of Allertop, a server for allergen prediction which is an alignment-free bioinformatics tool based on the ACC transformation of protein sequences into uniform equal length vectors followed by evaluation based on similarity in physico-chemical properties between the test protein and nearest neighbors. When analyzed individually by AllerTop server, all the eight chimeric Cry proteins showed the absence of allergenicity reiterating the earlier analyses of absence of allergenic potential in them.

Most of the dietary proteins that are ingested in the human gut are immediately exposed to hydrolytic digestion and/or degradation. However, an important character of allergenic proteins is the stability to the activity of digestive enzymes like pepsin etc. In silico as well as in vitro analysis can be used to delineate them based on the ability of pepsin to digest the proteins. In our study, two of the eight chimeric proteins, Cry1AcF and Cry1Aabc were further assessed using ExPASy-peptide cutter server to demonstrate the presence of pepsin digestibility sites [Additional file 1]. It was seen that the number of pepsin sites in the chimeric toxins were similar to that of the already analysed Cry1C and Cry1Ac toxins (igmoris.nic.in/files/Biosafety_data/Biosafety/metahelix) further confirming the non allergenicity of the chimeric cry proteins. This study is the first of its kind to use this tool and explicitly demonstrate the non-allergenic potential of the selected protein engineered toxins.

### In vitro assessment of the chimeric proteins for allergenic potential

An integral part of the safety assessment of GM food lies in the fool proof knowledge and demonstration that the transgene deployed in the crop is not a food allergen. Concomitant to the in silico analyses to establish this, various in vitro analyses are carried out as a part of risk assessment before judging it to be safe [31]. This involves a number of biochemical and toxicological studies as outlined by the biosafety regulators. Processing of the GM plant material prior to or after ingestion in the human gut is a vital aspect to be considered for toxicological safety assessment. Availability of the intact protein during absorption in the gut not only shows that it is heat stable but also depicts its resistance to the action of digestive enzymes. These parameters provide strong evidence to demonstrate the allergenic potential of proteins, as allergens are known to be both heat stable as well as resistant to digestion by the enzymes in the gut.

### Thermal stability of the chimeric proteins

**Fig. 2:**
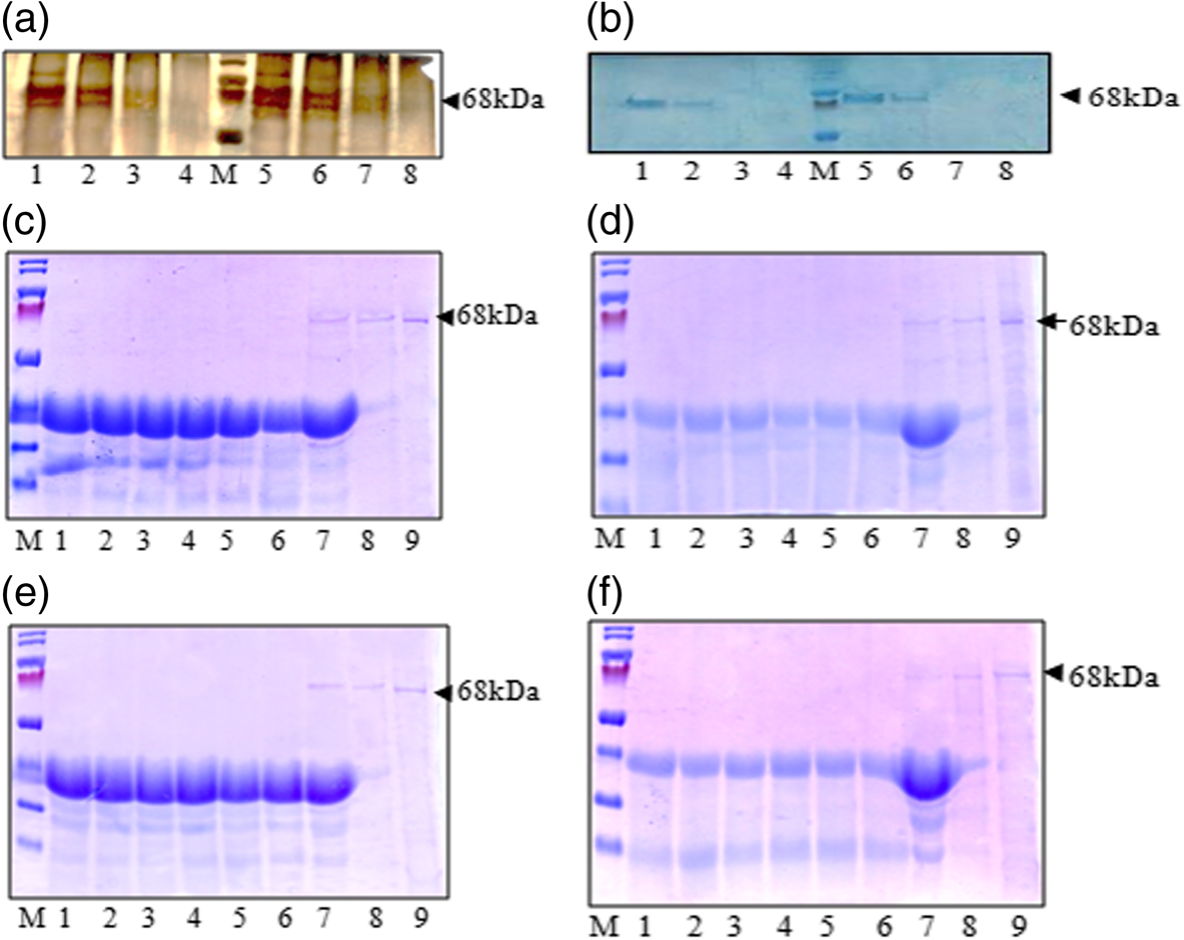
In vitro assessment of the chimeric proteins for allergenic potential. Analysis of the chimeric proteins for thermal stability: **a** and **b** SDS PAGE and Western blot analysis respectively showing stability of Cry1AcF and Cry1Aabc proteins after incubation at 100 °C for different time intervals. Lane 1- Cry1AcF without incubation, Lane 2–10 min incubation, Lane 3–30 min incubation, Lane 4–60 min incubation, M-Marker, Lane 5-Cry1Aabc without incubation, Lane 6–10 min incubation, Lane 7–30 min incubation, Lane 8–60 min incubation. Analysis of the chimeric proteins for pepsin digestibility: **c** and **d** SDS PAGE for in vitro digestibility of Cry1AcF protein in SIF (simulated intestinal fluid) with pepsin at pH 1.2 and 2.0 respectively and incubated at 37 °C for different time periods. **e** and **f** SDS PAGE for in vitro digestibility of Cry1Aabc protein in SIF with pepsin at pH 1.2 and 2.0 respectively and incubated at 37 °C for different time periods. (M- Marker, Lane 1–0 s incubation, Lane 2–30s incubation, Lane 3–60s incubation, Lane 4-5 min incubation, Lane 5–10 min incubation, Lane 6–30 min incubation, Lane 7- test protein with pepsin and without SIF and 30 min incubation, Lane 8- test protein with SIF and without pepsin and 30 min incubation, Lane 9- Intact test protein with 30 min incubation)

In the present study, two chimeric proteins, Cry1AcF and Cry1Aabc were chosen for the assessment of two important parameters, resistance to heat and the digestive enzyme pepsin. Incubation of the chimeric proteins at 100 °C for different time periods ranging from 0 to 60 mins demonstrated that both the chimeric proteins degraded completely after 10 mins (Fig. 2 a and b). Silver staining and western blot analysis of the assay mixtures corroborated the time of degradation indicating that the cry proteins could be degraded into simpler structures and therefore were safe for consumption.

### In vitro digestibility of the chimeric proteins with pepsin

Evaluation of the allergenic potential of proteins based on the digestibility by purified porcine pepsin is one of the widely used methodologies worldwide [31]. Porcine pepsin is an aspartic endopeptidase with broad substrate specificity and optimal activity between pH 1.2 and 2.0. A validated in vitro assay [26] that uses a fixed porcine pepsin: protein ratio and simulated intestinal fluid (SIF) under both pH 1.2 and 2.0 (as per the recommendations of FAO/WHO) was used in the present study. The assay demonstrated that both the chimeric Cry proteins were completely digested as soon as they were added into the assay mix (0 s) in both pH 1.2 as well as pH 2.0 because of the presence of active pepsin (Fig. 2 c–f). However, intact cry proteins were observed under conditions either lacking SIF or porcine pepsin indicating the susceptibility of the chimeric proteins to pepsin activity. This is in coherence with the observation that, following pepsin digestion, the non-allergenic food proteins were digested within approximately 30 s while major food allergens exhibited pepsin-stable fragments that were detectable even after 8–60 min.

## Conclusion

This study therefore reconfirms that the selected chimeric proteins encoded by the selected chimeric genes are of significance both towards scientific and safety perspective. Based on the comprehensive bioinformatic and supportive in vitro analyses, we demonstrated that the selected chimeric Cry proteins complied with parameters used to identify a protein as non-allergenic. Consumption of these proteins following deployment in transgenic crops will therefore not result in triggering of any allergenic response. The study also demonstrates the usefulness of protein engineering as an additional alternative to engineer insect resistance.

## Additional file

Additional file 1: In silico assessment of pepsin digestibility sites in Cry1AcF and Cry1Aabc using Expasy peptide cutter. (XLSX 32 kb)

## Abbreviations

ACC: Auto and cross-covariance
ARPs: Allergen representative peptides
*Bt*: *Bacillus thuringiensis*
DELTA-BLAST: Domain enhanced look-up time accelerated–BLAST
GM: Genetically modified
*K*NN: *k* nearest neighbors
PSSMS: Position specific score matrix
SVM: Support vector machine
